# Early Reverse Transcription Is Essential for Productive Foamy Virus Infection

**DOI:** 10.1371/journal.pone.0011023

**Published:** 2010-06-11

**Authors:** Alessia Zamborlini, Noémie Renault, Ali Saïb, Olivier Delelis

**Affiliations:** 1 CNRS UMR7212, Inserm U944, Université Paris Diderot, Institut Universitaire d'Hématologie, Paris, France; 2 Chaire de Biologie, Conservatoire National des Arts et Métiers, Paris, France; 3 LBPA, CNRS, ENS de Cachan, Cachan, France; Institut Pasteur, France

## Abstract

**Background:**

Although viral RNA constitutes the majority of nucleic acids packaged in virions, a late occurring step of reverse transcription leads to the presence of infectious viral cDNA in foamy virus particles. This peculiarity distinguishes them from the rest of the retroviral family.

**Principal Findings:**

To evaluate the respective contribution of these viral nucleic acids in the replication of foamy viruses, their fate was studied by real-time PCR and RT-PCR early after infection, in the presence or in the absence of AZT. We found that an early reverse transcription step, which occurs during the first hours post-entry, is absolutely required for productive infection. Remarkably, sensitivity to AZT can be counteracted by increasing the multiplicity of infection (moi). We also show that 2-LTR circular viral DNA, which appears as soon as four hours post-infection, is transcriptionally competent.

**Conclusion:**

Taken together, our data demonstrate that an early reverse transcription process, which takes place soon after viral entry, is indispensable for infectivity of FVs at low moi, when the amount of DNA-containing particles is not sufficient to lead to a productive infection. This study demonstrates a key role of the packaged viral RNA in the foamy virus infection, suggesting that the replication of this virus can be achieved by involving either viral DNA or RNA genome, depending on the condition of infection.

## Introduction

Foamy viruses (FVs) are complex animal retroviruses sharing homologies in comparison to the conventional replication pathway of retroviruses such as HIV-1. For example, it has been reported that primate foamy virus (PFV-1) integrase, which is responsible for the indispensable integration step of the viral genome into the host cell DNA, displays a new specific restriction-like cleavage activity on palindromic sequences. An analogous function has also been demonstrated for Human immunodeficiency virus type 1 (HIV-1) integrase [1]-[4]. However, Foamy viruses present several features that set them apart among this viral group [5]. For example, the Gag polyprotein of FVs, which is involved in the FV integration process, is not processed into matrix, capsid and nucleocapsid sub-domains as in most animal retroviruses, but instead is C-terminally cleaved by the viral protease [6]-[8], in a manner similar to the processing of yeast retrotransposon Ty1 Gag [9]. However, the most striking feature of FVs stands on their replication strategy, which resembles in some aspects to that of hepadnaviruses [10]. In particular, full-length infectious viral DNA is found in extracellular FV particles, demonstrating that part of viral genomic RNA is reverse transcribed after proviral integration in producer cells and incorporated into virions [10]–[12]. Consequently, whereas packaged viral DNA is less than 0.001% in the case of HIV-1 [13], it can represent as much as 20% of total viral nucleic acids in extracellular FVs, suggesting that viral DNA could be functionally important for productive infection [14]. Treatment of target cells with zidovudin (AZT) prior to infection had only a minor effect on FVs infectivity, strongly suggesting that incoming viral DNA is sufficient to support efficient replication [12]. However, the presence of a majority of viral RNA in virions raises concern about the biological role of these molecules in the replication cycle of FVs. Indeed, the presence of infectious viral DNA in extracellular virions does not exclude the existence of an early, post-entry, reverse transcription step. By quantitative real-time PCR, we demonstrated the existence of a biphasic viral DNA synthesis in target cells following infection, which was inhibited by AZT [15].

Here, the fate of viral DNA and RNA was studied during the early stages of PFV infection, in the presence or absence of AZT. Using real-time PCR and RT-PCR, we clearly demonstrated that an early reverse transcription step is absolutely required for productive PFV infection. Furthermore, we also reported that 2-LTR circles, which are described as dead-end products of retroviral replication, are actively transcribed as already shown for HIV-1 [16]. Taken together, our study demonstrates the importance of both nucleic acids (DNA and RNA) in foamy virus infection.

## Results

### Nucleic acid composition of PFV virions

**Figure 1:**
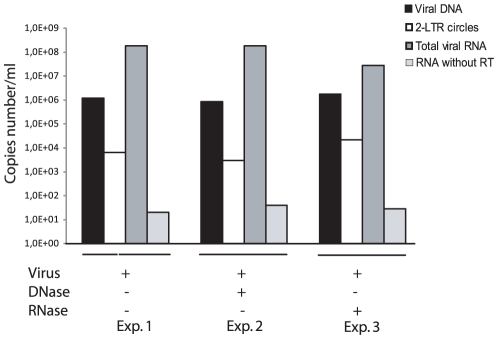
Characterization of the nucleic acid content of purified PFV particles. Copy numbers of DNA (linear and 2-LTR circles) and RNA were determined by real-time PCR and RT-PCR. Each quantification has been performed in triplicate. However, the standard deviation is too weak to be indicated. Viral supernatants were treated with DNase or RNase. The quantification of viral RNA without reverse transcription is indicated as a control. Results were expressed in copies per ml of supernatant.

Although RNA represents the vast majority of the nucleic acid incorporated in FV particles [12], [14], full-length viral DNA was detected in extracellular virions, representing between 10 and 20% of packaged nucleic acids [12], [14]. Since previous estimations were mainly based on semi-quantitative approaches [12], [14] or Southern-blot [12], quantitative PCR and RT-PCR were performed on cell-free virus stocks to precisely measure the viral DNA and RNA contents of extracellular particles (Fig. S1). For that purpose, viral supernatant from acutely PFV-infected FAB cells [17] was pelleted following sucrose gradient centrifugation as already described [15]. DNAse and RNase treatment were performed without permeabilization of viral particles in order to remove free nucleic acids. Following extraction of total genomic nucleic acids, all viral RNA forms were quantified by real-time RT-PCR using the TaqMan technology (see Table S1 for primers and probes sequences). RNA quantifications were also performed with *gag*-specific primers which gave the same results in comparison to those performed with primers and probes Spuma A, Spuma S and Spuma TM (data not shown). This observation suggests that the viral RNA extracted from purified FVs viral particles is a full-length RNA coding for the entire genome. This hypothesis has been reinforced by the functional role of this viral RNA (see next section). A very weak signal was obtained when the reverse transcription step was omitted, testifying the absence of viral DNA following RNA extraction (Fig. 1). Given these stringent controls, we confirmed that viral RNA represented the main nucleic acid detected in extracellular PFV particles (Fig. 1). Since a viral particle contains 2 copies of the viral RNA [18], we estimated that DNA-containing viral particles represented about 6% of total viral particles in our experimental settings, consistent with previous reports. To calculate the percentage of viral RNA in FVs viral particles, we divided the number of viral RNA copies from RNase treated particles over the viral DNA copies from the same amount of DNase-treated particle. Interestingly, LTR-LTR DNA junctions were also detected (as already reported [15]) in 0.3% of DNA-harboring particles virions. These observations raised the question of the potential role of RNA in the foamy virus replication strategy but also the origin of the LTR-LTR junction detected in virions.

### Evidence of an early reverse transcription step

To evaluate the role of the different viral nucleic acids species packaged in virions during viral replication, the fate of intracellular viral DNA and RNA was monitored during the first 24 hours post-infection of human U373-MG cells, at an MOI of 0.01. At different time points, cells were collected, washed thoroughly and treated with trypsin to eliminate viral particles that had not been internalized, as already reported [16].

**Figure 2:**
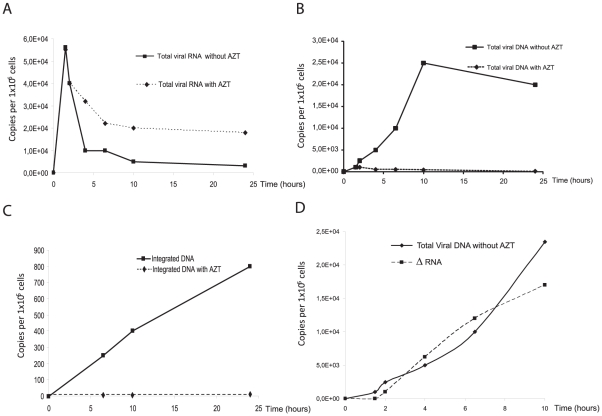
Kinetic analysis of PFV RNA and DNA synthesis. A representative experiment is shown. U373-MG cells were infected in the presence or absence of AZT (100 µM). The drug was added 2 hours before infection and kept on cells during the experiment. Levels of intracellular PFV DNA and RNA were monitored during the first hours of infection. (**A**) Dynamics of viral RNA with and without AZT monitored by real-time RT-PCR. Synthesis and fate of total viral DNA (**B**), integrated DNA (**C**) monitored by real-time PCR. Viral RNA involved in the reverse transcription step as well as viral DNA synthesis were monitored (**D**). At each time point post-infection, quantification of viral RNA copies without AZT is subtracted from viral RNA copies with AZT.

Since AZT is the only efficient inhibitor of FV RT [19], target cells were treated or not with this compound (AZT 100 µM) prior and during infection. We checked that, at the experimental settings (MOI = 0.01), AZT efficiently blocked viral replication as demonstrated by X-Gal staining of infected cells, as already reported [15]. A fast increase in intracellular viral RNA content was observed in the first hours post-infection (hpi), both in the presence or absence of AZT, most likely corresponding to incoming viral RNA. This result demonstrates that AZT treatment did not interfere with viral entry (Fig. 2A). Viral RNA content reached a maximum of 5.5×10^4^ copies per 10^6^ cells one hour post-infection. This was immediately followed by a slow decrease to reach a plateau at 2×10^4^ copies per 10^6^ cells and 0.5×10^4^ copies per 10^6^ cells, in the presence or in the absence of AZT, respectively (Fig. 2A). In three independent experiments, viral RNA content in infected cells after viral entry was always higher when cells were AZT-treated.

On the other hand, viral DNA content, in untreated cells, increased until 10 hpi and next declined until 24 hpi to reach 2×10^4^ copies per 10^6^ cells (Fig. 2B). This decrease is certainly a multi-parameter process due to the dilution of viral nucleic acids following cell division but also to the intrinsic stability of viral DNA in the infected cell, as already reported for HIV-1 [20]. In the presence of AZT, a faint signal was detected in the first hpi reaching 1.2×10^3^ copies per 10^6^ cells, which likely represented incoming packaged viral DNA [14]. However, under these settings, the DNA content rapidly reached a plateau at 500 copies per 10^6^ cells (Fig. 2B).

Integrated PFV DNA was quantified by Alu-LTR real-time PCR as already described [21]. Proviral DNA was detected as soon as 6 hpi, as already reported for HIV-1 [16], and increased significantly to reach a maximum of 800 copies per 10^5^ cells, representing about 40% of total viral DNA content at 24 hpi (Fig. 2C). As expected, no integrated viral DNA could be detected in the presence of AZT, even at late time points (data not shown). To normalize the viral RNA quantification we chose the cyclophilin A mRNA that was reported to be unaffected by various drugs or viral infection [22]. Assuming that *de novo* transcription did not occur at least during the first 10 hpi, as demonstrated for other retroviruses [16], [23], the curve standing for viral RNA content under AZT treatment reflected the intrinsic stability of incoming viral RNAs in the infected cell since DNA synthesis is inhibited. In contrast, the curve representing the RNA content in the absence of AZT resulted from two concomitant processes, i.e. the intrinsic stability of viral RNAs in the cytoplasm and their involvement in an early reverse transcription step explaining the fact that higher amounts of viral RNA are detected in the presence of the RT inhibitor, in a reproducible manner. The quantity of viral RNA contributing to reverse transcription can thus be estimated as the difference between the viral RNA content under AZT treatment and that measured in untreated cells. Remarkably, at each time point, the number of viral RNA molecules that disappeared correlated with the number of DNA molecules synthesized (Fig. 2D). Altogether these observations demonstrated the existence of an early reverse transcription step.

### Stability of viral nucleic acids

**Figure 3:**
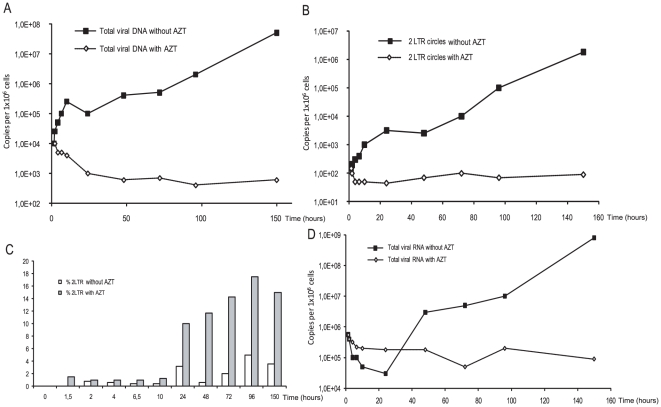
Kinetic analysis of PFV RNA and DNA synthesis over 6 days post-infection. U373-MG cells were infected in the presence or absence of AZT. Viral DNA (**A**), 2-LTR circles (**B**), viral RNA (**C**) were monitored by real-time PCR and RT-PCR. The percentage of 2-LTR circles over linear viral DNA is represented (**C**).

To examine stability of viral DNA and RNA, U373-MG cells were infected at a MOI of 0.1 in the absence or presence of 100 µM AZT (Fig. 3). We monitored levels of viral DNA forms as well as viral RNA over a 6 days period. Viral nucleic acids were quantified from PFV-infected cell extracts. In the presence of AZT, viral DNA decreased to reach a steady state of 10^3^ copies per 10^6^ cells (Fig. 3A). In the absence of AZT, we observed a strong viral DNA synthesis due to the spread of the viral replication. Interestingly, we are able to detect viral DNA until 6 days post-infection. Furthermore, 2-LTR circles also decreased to reach a steady state of 10^2^ copies per 10^6^ cells (Fig. 3B). Consequently, in this condition, the percentage of 2-LTR circles is increased in the presence of AZT to reach 16% of total viral DNA (Fig. 3C). Monitoring of viral RNA synthesis demonstrate strong activity of transcription in the absence of reverse transcription inhibitor (Fig. 3D). In the presence of AZT, viral RNA was still detected. This could result from the transcriptional activity of the incoming viral DNA or the remaining incoming viral RNA. However, no integrated viral DNA is detected in this condition. These data reinforce the idea of the transcriptional activity of unintegrated PFV viral DNA as demonstrated in HIV-1 infection [16].

### Transcriptional activity of 2-LTR circles

**Figure 4:**
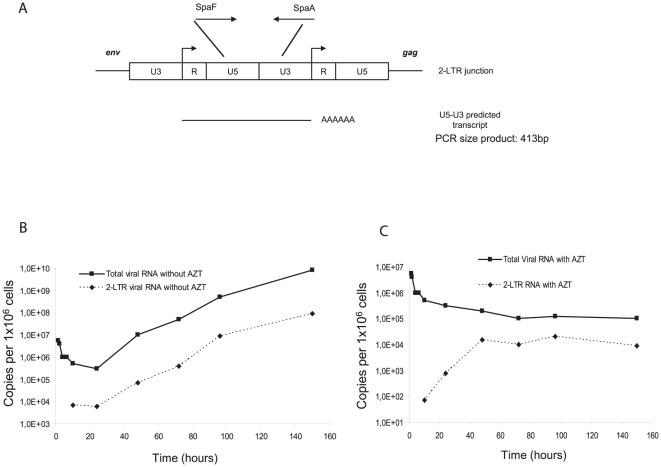
Transcriptional activity of 2-LTR circles. Primers used for the quantification of U5-U3 transcript are indicated. (**A**). The U5, R, U3 regions of the 2-LTR circles are represented. Arrowheads indicate transcription start sites. Kinetics of total viral RNA as well as U5-U3 transcript content were evaluated during a multiple round of infection of U373-MG cells in the absence (**B**) or presence of AZT (**C**).

Integrated viral DNA is not the only viral nucleic acid competent for viral gene expression [24]. Indeed, unintegrated viral DNA can also sustain efficient transcription [25], [26], as it has been recently reported for 2-LTR circles during HIV-1 replication [16]. Consequently, viral RNA synthesized from the upstream R region contains the U5-U3 junction (Fig. 4A). We assessed whether such circular viral molecules could generate LTR-LTR transcripts during PFV replication. For that purpose, we designed a RT-PCR assay with the same primers used to measure the LTR-LTR DNA junction. Synthesis of U5-U3 transcripts was initially detected 10 hpi and increased regularly, reaching a peak 150 hpi with 5×10^7^ copies per 10^6^ cells (1% of total viral RNA) (Fig. 4B). The U5-U3 transcripts raised to the same extent as the total viral RNA during the course of infection (Fig. 4B). Following addition of AZT at 10 hpi, U5-U3 transcripts were still detected in infected cells, representing 10% of total viral RNA content (Fig. 4C). The over-representation of U5-U3 mRNA under these settings correlated to that of 2-LTR circles (data not shown and [15]) suggesting that they reflected transcripts that are expressed from circular forms of viral DNA.

### Sensitivity to AZT depends on virus dose

**Figure 5:**
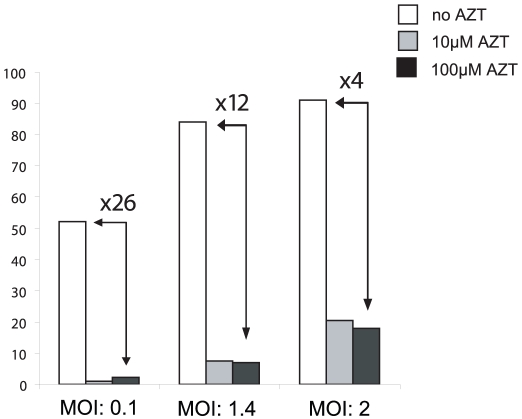
Effect of increasing MOI on infectivity under AZT treatment. Indicator FAG cells were infected with increasing MOIs in the presence or absence of AZT at two different concentrations, 10 µM and 100 µM. Forty eight hpi, GFP expression was monitored by flow cytometry. The values represented the average of three independent experiments.

Absence of proviral integration and DNA synthesis under AZT treatment demonstrated total inhibition of FV replication. This finding, which confirmed our previous observations [15], is in contrast with other reports showing that AZT has low to weak effect on FV replication when added during infection [11]. One hypothesis to explain this discrepancy relies on distinct viral DNA contents of virus stocks. To assess this possibility, BHK21GFP-indicator cells (called FAG cells [27]) were infected with increasing MOI of PFV (raising therefore the amount of incoming viral DNA) in the presence of AZT. Since FAG cells stably harbor the GFP reporter gene under the control of the PFV LTR, infection will lead to GFP expression, which is easily measured by flow cytometry 48 hpi. In the absence of AZT, GFP-positive cells represented 52, 84 and 92% of the cell culture following infection with a MOI of 0.1, 1.4 and 2, respectively. In the presence of the RT inhibitor, 2% of GFP-positive cells were detected at MOI 0.1, confirming previous results [15]. This percentage reached 20% at higher MOI (Fig. 5), demonstrating that sensitivity to AZT depended on the initial viral stock input, and therefore the amount of incoming viral DNA.

## Discussion

In this report, using real time RT-PCR and PCR assays, we confirmed the presence of a majority of viral RNA in extracellular FVs particles. Moreover about 6% of virions released from acutely infected cells contained viral DNA, including 2-LTR circles. Studying the fate of incoming viral nucleic acids in PFV-infected cells, we confirmed the existence of an early viral DNA synthesis step, which was abrogated under AZT treatment. Remarkably, the kinetics of viral DNA synthesis correlated with the decrease in viral RNA content, demonstrating for the first time the importance of an early reverse transcription step for FV replication.

Previous reports demonstrated that FV reverse transcription occurred late in the replication cycle [10]-[12], leading to the presence of full-length viral DNA in extracellular particles. DNA extracted from these particles can lead to virus production after transfection [10]. Consequently, treatment of cells by AZT prior to infection did not drastically affect viral replication. However, careful examination of previous reports clearly shows that AZT did not totally abrogate FV replication. Moebes and *al.* demonstrated, in the case of BHK-21 infection, that the titer of pcHSRV2 virus was 3.63×10^3^/ml in the absence and 9.5×10^1^/ml in the presence of AZT, representing a decrease of 36-fold decrease of infectivity in the presence of the inhibitor [11]. Our data demonstrate that viral RNA is also infectious and clearly show that sensitivity to AZT is dependent on the initial amount of incoming viral DNA. At a low MOI, the ratio of infected cells between treated and untreated conditions is 26 but only 4 when the MOI is 2. Therefore, we suggest that incoming viral DNA can overcome the inhibitory effect of AZT at higher MOI. Based on previous reports and on the present study, we propose that FVs can use two distinct replication strategies depending on the target cell, as already suggested [28]. The first one requires a step of early reverse transcription whereas the other bypasses this stage thanks to packaged infectious viral DNA. Infection process depends on the nature of the incoming viral nucleic acid in the infected cell.

Under our settings, we demonstrate that, even a high concentration of AZT, viral genome is detected even 6 days post-infection. Unintegrated viral DNA is described to have a short half life in infected cells but quite stable in quiescent cells [29], [30]. Detection of viral DNA could be due to a basal replication due to the inefficiency of the compound to prevent totally the reverse transcription process. Thus, a weak integration could occur and be at the origin of viral replication. A second hypothesis could involve the infectious viral DNA packaged in the virions. Further investigations are needed to conclude between these two hypotheses. However, in these settings, the percentage of 2-LTR circles is increased, consistent with a stronger stability of 2-LTR circles over linear unintegrated viral DNA [31]. Integrated viral DNA is described to be the sole template for efficient viral transcription. However, it has been demonstrated that human macrophages support persistent transcription from unintegrated HIV-1 DNA [32] highlighting the role of unintegrated viral DNA. In this report, we found a greater stability of 2-LTR circles over linear viral DNA in the case of PFV infection associated with specific transcription from these episomes. The precise role of 2-LTR circles, detected in viral particles, in the PFV replication needs to be studied because of the presence of infectious viral genome in PFV virions. Detection of such molecules in viral particles could be due to the endogenous reverse transcription or packaging of 2-LTR circles DNA in particles. Taken together, our data highly suggest that the two nucleic acids contained in PFV viral particles are able to lead to viral infection.

## Materials and Methods

### Cell culture and virus stocks

U373-MG, FAG and FAB cells (harbouring the HFV LTR-β-Gal construct [17]) were cultured in Dulbecco's modified Eagle medium with 10% foetal calf serum. For FAB cells, 1 µg/ml of G418 (Sigma, Saint Quentin Fallavier, France) was added. Cell-free virus stocks were titrated on FAB cells as described [17]. In some experiments, cells were treated with 100 µM AZT (SIGMA, Saint Quentin Fallavier, France). For pre-treatment experiment, AZT was added in the medium 2 hours before infection.

### Virus and nucleic acid purifications

Cell-free supernatant from acutely infected FAB cells was mixed with NTE buffer (100 mM NaCl, 10 mM Tris-HCl pH 7.4, 1 mM EDTA) placed on a 20% sucrose cushion and centrifuged (25,000 rpm, 3 hours, 4°C) using a SW41 rotor. Pellets were resuspended in PBS and aliquots were treated, or not, with 450 units of DNAse (Gibco, Cergy Pontoise, France) for 5 hours at 37°C or with Rnase A (40 µg/ml) during two hours at 37°C. DNA was extracted using the DNA Blood Mini kit (Qiagen, Courtaboeuf, France) and RNA was extracted using the RNeasy mini kit (Qiagen), according to the manufacturer's instructions. For analysis of RNA contained in virions, 5×10^6^ U373-MG cells were incubated with 200 µl of viral supernatant to act as a RNA carrier and lysed immediately by adding 600 µl of RLT buffer with β-mercaptoethanol (Qiagen). Then, RNA was recovered according to the manufacturer's instructions in 50 µl RNase-free water.

### Real-time PCR quantification

Amplification was performed by real-time PCR using the Light Cycler Instrument (Roche Diagnostics, Meylan, France) and measurements were performed using the Light Cycler quantification software version 3.5 (Roche Diagnostics). Sequences of all primers used for DNA and RNA quantifications are given in Table S1. Total PFV DNA was measured using primers in the *gag* gene described previously [15]. Two-LTR circles were amplified using primers spanning the LTR-LTR junction. These amplifications were realised in a 20 µl reaction volume containing 1X Light Cycler Fast Start DNA Syber Green Technique (Roche Diagnostics), 3.5 mM MgCl2, and 500 nM of each primer. The pHSRV13 plasmid harbouring the entire HFV genome was used for calibration of total viral DNA quantification. For 2-LTR circles quantification, the pR/U3 plasmid, obtained by cloning this 413 bp PCR product, was used for calibration. Integrated HFV DNA quantification was performed by using an *Alu-*LTR nested PCR as described previously [21]. Briefly, in the first-round PCR, *Alu*-LTR sequences, derived from integrated proviruses, were amplified in a 20 µl reaction mixture containing 1x Light Cycler Fast Start DNA Master Hybridation probes (Roche), 4 mM MgCl_2_, 100 nM of forward primer LambdaT-SpA and 300 nM of primers Alu 1 and Alu 2. Nested PCR was performed on 1/10 of the first-round PCR products in 1x Light Cycler Fast Start DNA Master Hybridisation probes, 4 mM MgCl_2_, 300 nM of forward primer LambdaT, 300 nM of primer Nested R and 200 nM of each hybridisation probes Sp FL and Sp LC (Table S1). To quantify integrated HFV copies a standard curve generated by the concomitant two-rounds PCR amplification of serial dilutions of the standard U373-MG chronically infected cells. We determined by serial dilutions of DNA and by Southern Blotting (data not shown) that viral DNA was exclusively present as integrated forms in cells. To control linear amplification arising from LambdaT-SpA primer, we performed a whole PCR protocol omitting *Alu* primers in the first round of PCR. Copy number of proviral DNA in each sample is determined by subtracting integrated DNA quantification in absence of *Alu* primers from copy number measured in presence of *Alu* primers. All results were reported for an equivalent number of cells by quantification of the β-globin gene (2 copies per diploid cell) using commercial available materials (Control kit DNA, Roche Diagnostics). All PCR conditions are given in Table S1.

### Quantification of viral and cellular RNA

Quantifications of viral and cellular RNA were performed by using real-time RT-PCR protocol. Amplification of viral RNA was achieved by using the TaqMan technology (Fig. 1D). Two-LTR transcripts as well as Cyclophilin A RNA were performed using Light Cycler Hybridisation probes. Sequences of primers and probes are given in Table S1. All RT-PCR are performed in 1x Light Cycler RNA Master Hybridisation probes (Roche Diagnostics), 3.25 mM Mn(OAC)_2_ and 500 nM of each primers and 200 nM of probes. RT-PCR cycle conditions are given in Table S1. Quantification of viral and cellular RNA were determined in a reference to a standard curve prepared by serial dilutions of an *in vitro* transcribed RNA (RiboMAX Large Scale RNA production system, Promega, Charbonnières, France) containing matching sequences. Normalization of results was performed measuring Cyclophilin A RNA. RT-PCR protocol was performed by hybridation probes technology using a standard curve generated by serial dilutions of RNA prepared by an *in vitro* transcribed RNA.

## Supporting Information

Table S1 Primer, probe sequences and PCR and RT-PCR cycle conditions.
(0.26 MB PDF)

Figure S1 Real-time PCR and RT-PCR strategies for the PFV-1 RNA and DNA quantification. Primers used to quantify total viral DNA (A) are located in the gag gene. Primers spanning the LTR-LTR junction were used to detect 2-LTR circles (B). Integrated DNA (C) is measured by two rounds of PCR as described previously [21]. Viral RNA species are depicted (D). Primers used in the RT-PCR strategy monitored all viral RNA species.
(1.27 MB EPS)
