# Supplementary material for: Early Reverse Transcription Is Essential for Productive Foamy Virus Infection
Source: PLoS One. 2010 Jun 11;5(6):e11023. doi: 10.1371/journal.pone.0011023 (PMC2884000; doi:10.1371/journal.pone.0011023)
Supplement: Table S1 — Primer, probe sequences and PCR and RT-PCR cycle conditions. (0.26 MB PDF) [file pone.0011023.s001.pdf]

Table S1: Primer, probe sequences and PCR and RT-PCR cycle conditions.

Primer or Probe	Sequence	Target
SpuIN F	5’-GGACCTGTAATAGACTGGAA-3’	Total viral DNA PCR size product:551bp
SpuR	5’-ATTTGCAGGTCTAATACTCTCC-3’	
SpA	5’-TAGTATAATCATTTCCGCTTTCG-3’	2 LTR circles and U5-U3 RNA PCR size product: 413bp
SpF	5’-CAATAAACCGACTTGATTCGAG-3’	
SpFL*	5’-GAGAGACACAAGGTTCTTAAATTGTCCTCATTCGC-3’[a]	
SpLC*	5’-ACTCCCTCTGACATCCAACGCTGGGCTAC-3’[b]	
LambdaT-SpA	5’-ATGCCACGTAAGCGAAACTTAGTATAATCATTTCCGCTTTCG-3’	Integrated viral DNA (first round)
Alu 1	5’-TCCCAGCTACTGGGGAGGCTGAGG-3’	
Alu 2	5’-GCCTCCCAAAGTGCTGGGATTACAG-3’	
LambdaT	5’-ATGCCACGTAAGCGAAACT-3’	Integrated viral DNA (second round) PCR size product: 234bp
Nested R	5’-GAAACTAGGGAAAACTAGG-3’	
SpFL*	5’-GAGAGACACAAGGTTCTTAAATTGTCCTCATTCGC-3’[a]	
SpLC*	5’-ACTCCCTCTGACATCCAACGCTGGGCTAC-3’[b]	
Spuma S	5’-CAAGGTTCTTAAATTGTCCTCATTC-3’	Total viral RNA PCR size product: 101bp
Spuma A	5’-TTTCCGCTTTCGGTGACCA-3’	
Spuma TM*	5’-ACTCCCTCTGACATCCAACGCTGGGC$_X$T-3’[C]	
CYC F	5’-CATCTGCACTGCCAAGACTGAG-3’	Cyclophilin A
CYC R1	5’-AGGGAACAAGGAAAACATGGAA-3’	
CYC FL*	5’-CCTCCACCCCATTTGCTCGCAGTA$_X$-3’[c]	
CYC LC*	5’-CCTAGAATCTTTGTGCTCTCGCTGCAGT-3’[b]	

***:** Probe sequence. A: modified probe with a 3’ fluorescein. B: modified probe with LC red 640 dye at the 5’ end and phosphorylated at the 3’ end. C: modified probe with 6-carboxyfluorescein (FAM) at the 5’ end and phosphorylated at the 3’ end. X: 5-carboxytetramethylrhodamine group. Primers and probes were purchased from TIB MOLBIOL (Berlin, Germany).

Target	RT	Denaturation	PCR cycles
Total viral DNA	none	95°C 10min	(95°C 10s, 60°C 10s, 72°C 30s)x50
2 LTR circles	none	95°C 10min	(95°C 15s, 58°C 10s, 72°C 20s)x50
Integrated viral DNA (first round)	none	95°C 8min	(95°C 10s, 60°C 10s, 72°C 170s)x15
Integrated viral DNA (second round)	none	95°C 8min	(95°C 10s, 63°C 10s, 72°C 8s)x50
Total viral RNA	61°C 25min	95°C 4min	(95°C 15s, 60°C 60s)x50
U5-U3 RNA	61°C 25min	95°C 4min	(95°C 1s, 58°C 10s, 72°C 20s)x50
Cyclophilin A	61°C 25min	95°C 4min	(95°C 1s, 56°C 10s, 72°C 15s)x50
